# Recurring desmoid tumor of the neck: A case report

**DOI:** 10.1002/ccr3.7227

**Published:** 2023-04-16

**Authors:** Kipras Pribuišis, Lukas Vaidelys, Julius Piluckis, Evaldas Padervinskis, Saulius Vaitkus, Valdas Šarauskas

**Affiliations:** ^1^ Department of Otolaryngology Academy of Medicine, Lithuanian University of Health Sciences Kaunas Lithuania; ^2^ Department of Pathology, Academy of Medicine Lithuanian University of Health Sciences Kaunas Lithuania

**Keywords:** desmoid tumor, perivertebral space, recurrence

## Abstract

The infiltrative growth pattern of desmoid tumors and their proximity to important anatomical structures make them difficult to manage. Mutilating surgery should be avoided, while surveillance or radiotherapy remain valid options.

## The infiltrative growth pattern of desmoid tumors and their proximity to important anatomical structures make them difficult to manage. Mutilating surgery should be avoided, while surveillance or radiotherapy remain valid options.

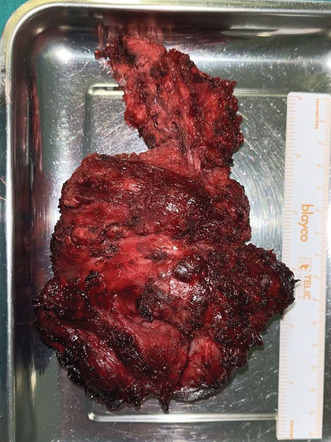

## 1 INTRODUCTION

Desmoid tumors (DTs), also histologically known as desmoid‐type fibromatosis (DTF), are rare, locally invasive, benign lesions with no metastatic potential that often prove challenging to manage due to their infiltrative growth pattern. We present a salvage case of a 20‐year‐old male patient with a DT managed by resection and radiotherapy and the results of subsequent follow‐ups.

DTs can affect any body part but are more common in the extremities, abdominal wall, and mesentery.^1^ Only 7%–10% of DT manifests in the head and neck region.^2^ The mean age of diagnosis is around 33 years, presenting two to four cases per million yearly, and tends to be less common in males.^3^, ^4^

Histologically, DT are monoclonal proliferations of benign‐appearing fibroblasts often characterized by locally infiltrative growth.^1^ The disruption in the Wnt/β‐catenin pathway is considered the critical component in the pathogenesis of DT.^5^, ^6^ The Wnt pathway and the adenomatous polyposis coli (APC) gene regulate intra‐cellular β‐catenin levels.^5^ A non‐functional APC gene results in excessive accumulation of intracellular β‐catenin. In turn, high levels of β‐catenin may cause overstimulation of genes resulting in excessive cell proliferation and differentiation, manifesting as DTF. Mutations in other genes, such as AKT1, BRAF, CTNNB1, and TP53, have also been reported in DTF.^7^ This genetically determined excessive benign cell proliferation combined with a margin‐negative resection (R0) being unlikely due to its infiltrative growth pattern, lack of capsule, and common localization near critical structures may be one of the causes of DT having a high probability of recurrence after surgery.^2^, ^3^, ^8^, ^9^ We present a salvage case of a DT located in the left posterior part of the neck managed by resection followed by radiotherapy.

## 2 CASE PRESENTATION

A 20‐year‐old male patient with a neck mass on the left side was referred to our department. The neck mass has increased for the last 3–4 months. The patient complained of a fever in the evenings, an increased neck mass size, and loss of appetite and weight for the past 3 months.

**FIGURE 1:**
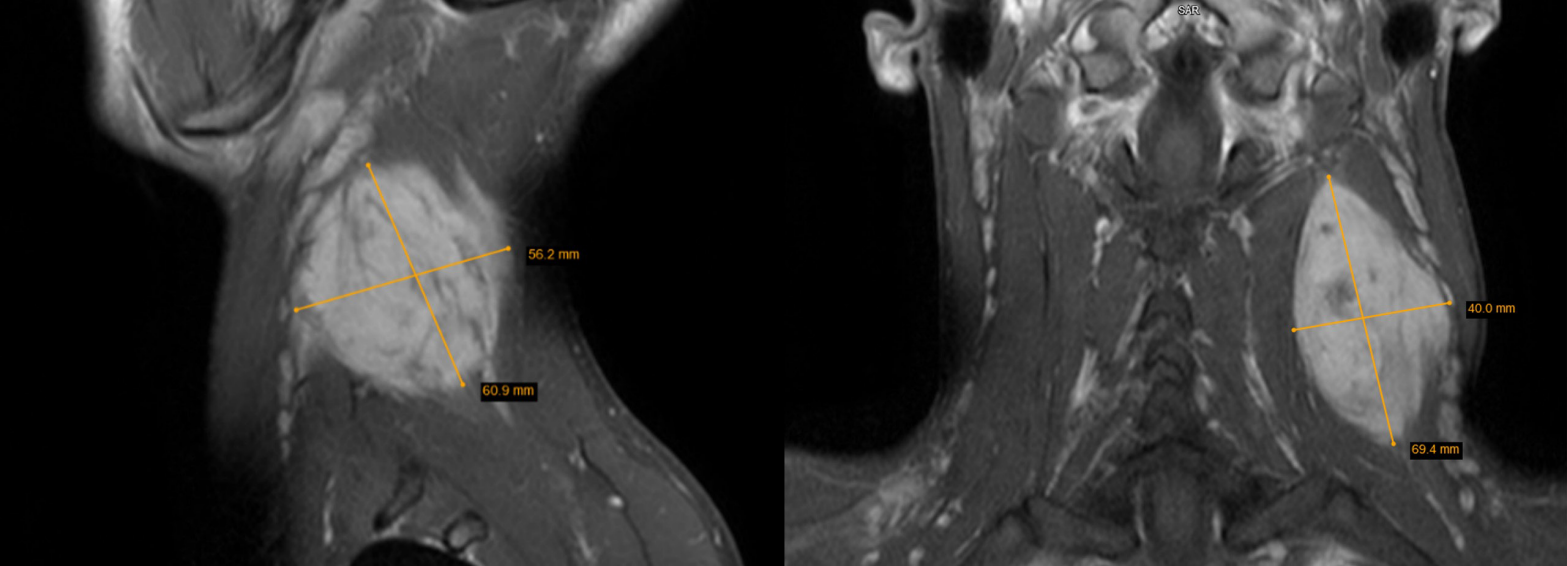
Magnetic resonance imaging (left—sagittal view; right—coronal view) of a mass in the paraspinal compartment of the perivertebral space measuring 69 × 40 × 56 mm.

During the initial workup, neck asymmetry was observed. The immobile, tender neck mass was palpated on the left side along with the sternocleidomastoid muscle. Magnetic resonance imaging (MRI) with intravenous contrast revealed a heterogeneous mass in the paraspinal compartment of the perivertebral space measuring at around 69 × 40 × 56 mm (Figure 1). Ultrasound‐guided needle biopsy histology results were DTF.

Surgical removal was attempted—the histopathology result: DTF, positive margins (R1). An active observation was considered, but contact with a patient was lost after discharge.

**FIGURE 2:**
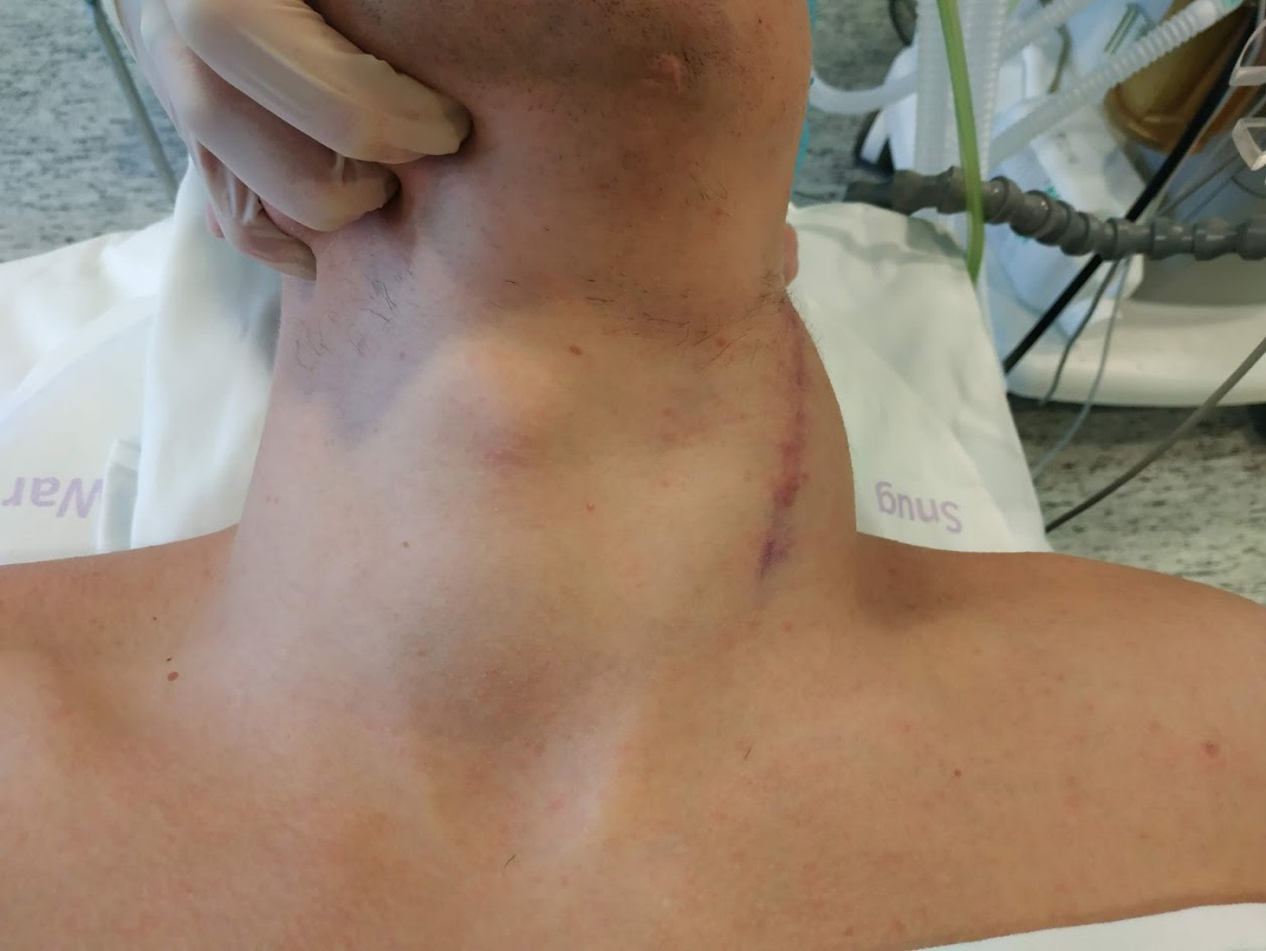
Frontal view of the patient's neck before secondary tumor dissection.

Ten months after the initial surgery, the patient contacted the department with complaints of a tender neck mass that was increasing in size. Neck movements were restricted (Figure 2).

**FIGURE 3:**
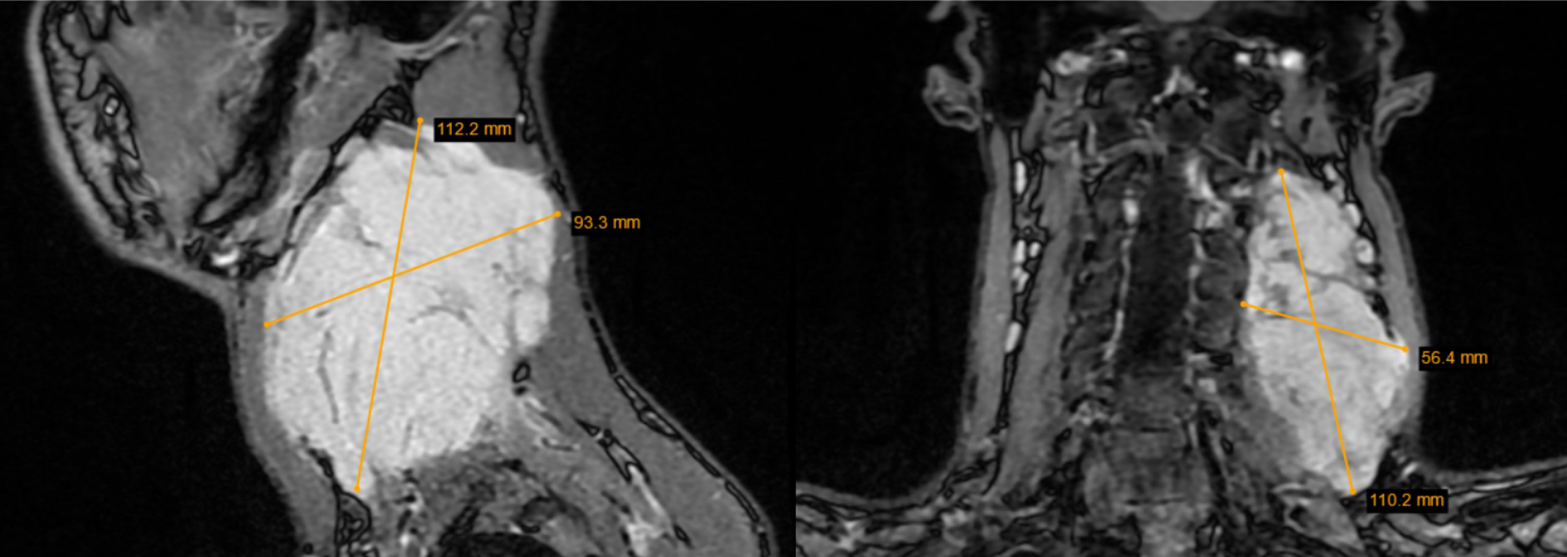
Magnetic resonance imaging (left—sagittal view; right—coronal view) of a recurrent tumor of the perivertebral space measuring 110 × 56 × 93 mm.

MRI with intravenous contrast displayed a polycyclic tumor of the perivertebral space 110 × 56 × 93 mm in size (Figure 3). The tumor had infiltrated parts of the dorsolateral muscles. The structures of the carotid space were dislocated, but no apparent infiltration was observed in the MRI.

The patient was discussed with a multidisciplinary head and neck team—radical resection was unlikely due to the tumor infiltration into adjacent muscles. Still, given the patient's young age, pressure on the carotid space structures, and possible long‐term effects of radiation, surgical debulking followed by adjuvant radiotherapy was advised.

**FIGURE 4:**
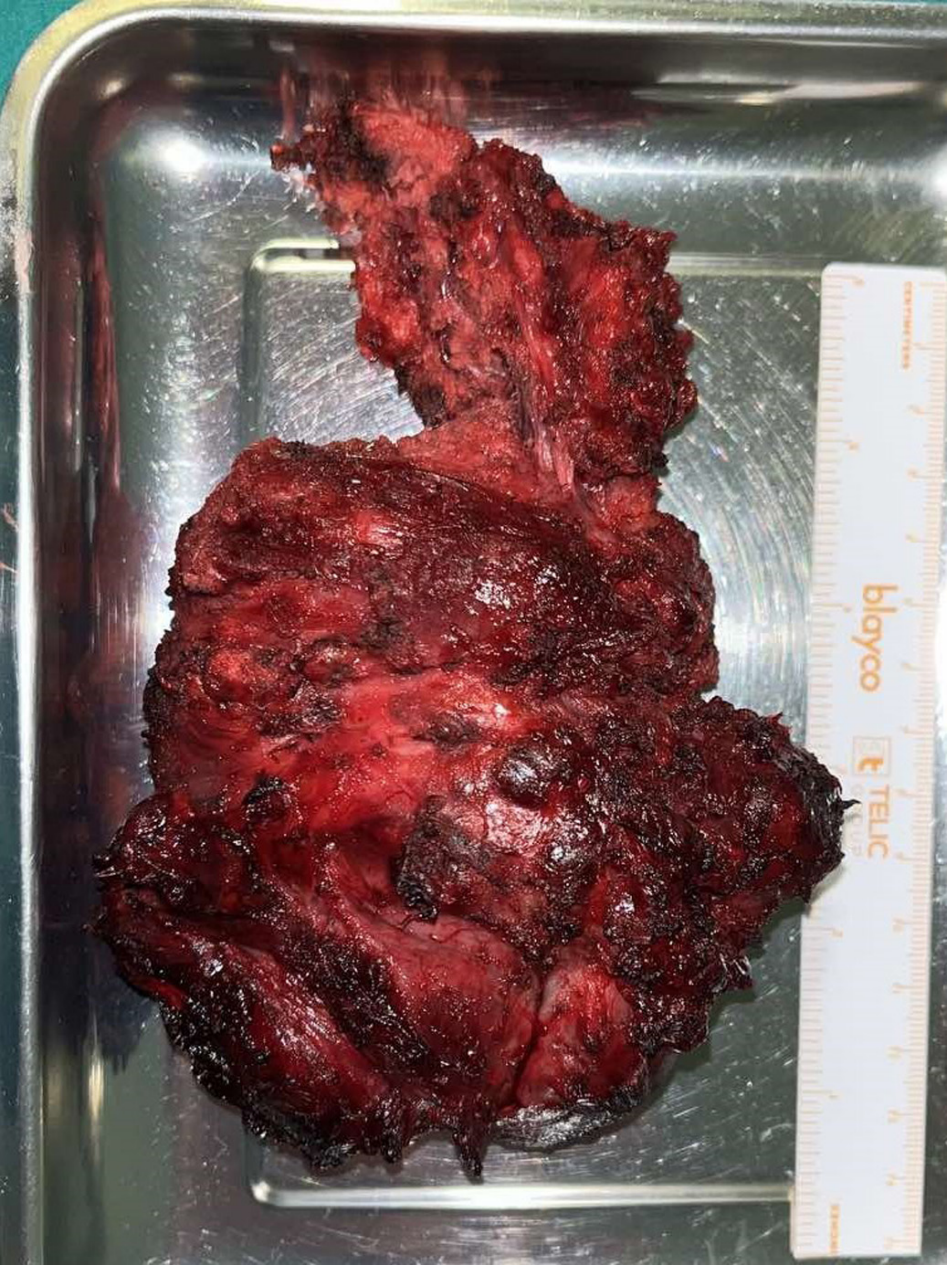
The dissected neck tumor measuring in at 90 × 55 × 35 mm.

**FIGURE 5:**
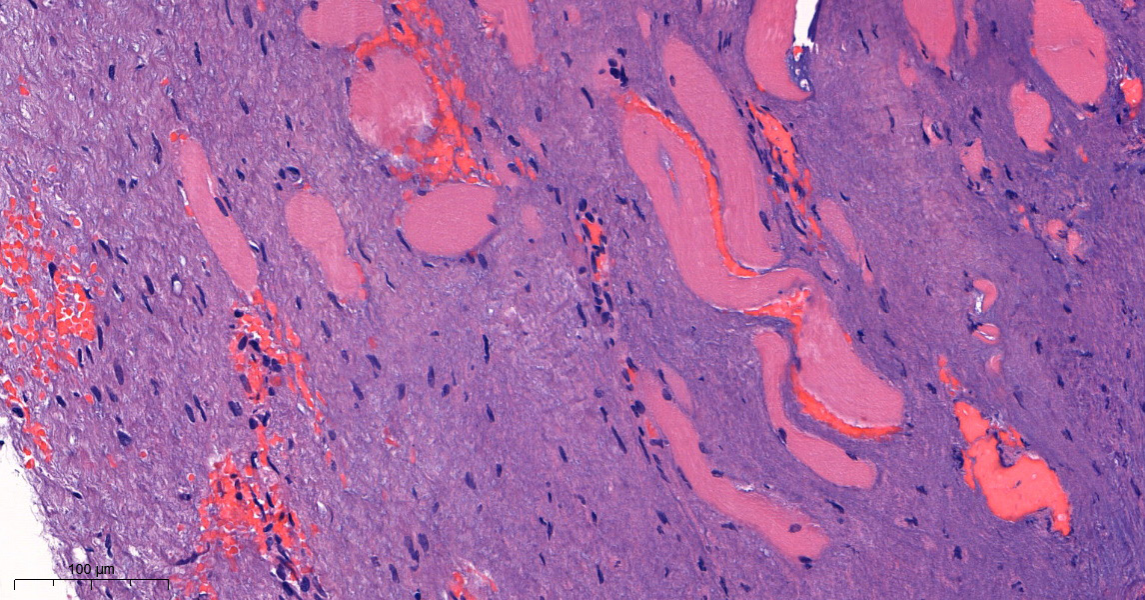
A 100 μm microscopic view of the desmoid tumor depicting sparsely cellular, fibrous tumor tissue with sparse fibroblasts spread around striated muscle cells – typical for the desmoid‐type fibromatosis.

The debulking surgery was performed through a Z‐shaped incision. The tumor was dissected while preserving the vagal, marginal, accessory, and brachial plexus nerves. The jugular vein, sternocleidomastoid, and anterior‐upper part of the trapezius muscles were removed due to the tumor invasion (Figure 4). As expected, the histopathological report resulted in DTF and positive margins (R1). Tumor cells react positively with beta‐catenin immunostaining. A cellular, poorly demarcated tumor spreading between adipose tissue and striated muscles, formed by mitotically inactive spindle‐shaped cells with an oval nucleus and eosinophilic cytoplasm, can be observed in Figure 5.

Gene mutations of APC, TP53, NF2, and other desmoid‐type genes were absent. The postoperative period was uneventful, with the patient having minimal sensory and no motor loss in the face, neck, and left arm. One month after surgery, 60 Gray radiotherapy was applied to the affected site. No radiation‐induced dermatitis was observed.

On follow‐up, 5 months after completing radiotherapy, the patient presented with slightly weaker lateral head movement, lateral shoulder abduction above 90 degrees, and intermittent numbness of the left fingers. No clinical signs of recurrence were observed. The tumor was indistinguishable from the surrounding anatomical structures in MRI after radiotherapy.

## 3 DISCUSSION

The lack of metastatic ability in DT often leads to a good patient survival prognosis. Some newly diagnosed and asymptomatic DT may achieve up to 50% long‐term stability or even spontaneous regression.^10^, ^11^, ^12^ However, DT's unpredictable behavior or cosmetic defects caused by DT traditionally require surgical resection. The tendency for infiltrative growth, lack of capsule, and proximity to critical anatomical structures pose a significant challenge for complete surgical removal in the head and neck region.^2^, ^3^, ^8^, ^9^ The effort to avoid mutilating or debilitating surgery often leads to positive margins and a high local recurrence rate.

Active observation for slow‐growing tumors should be considered before and after surgery, even if the negative resection margin is achieved.^9^, ^11^ In case of rapid recurrence, a timely re‐evaluation followed by secondary resection can be performed. A secondary resection should be as thorough as possible without causing disability or mutilation to the patient.^8^ Large initial tumor size, young patient age, and tumors located in the extremities or mesentery have been associated with increased postoperative recurrence.^13^, ^14^ Since total surgical excision would lead to mutilation in most cases, radiotherapy can be used for further treatment in individual patients. Surgery with radiotherapy was reported to be more effective than surgery alone (risk of recurrence 22% vs. 54%), with the difference being statistically significant.^9^ Furthermore, radiation‐induced morbidity and long‐term effect should be considered and kept as low as reasonably possible, especially when applied to young patients.^11^

## 4 CONCLUSION

The infiltrative growth pattern of DTs and their proximity to important anatomical structures make them challenging to manage. Mutilating surgery should be avoided, while active surveillance remains a valid option when growth is slow and critical structures are at a distance. Radiotherapy can be a viable adjuvant treatment.

## AUTHOR CONTRIBUTIONS

**Kipras Pribuišis:** Conceptualization; data curation; funding acquisition; methodology; resources; supervision; writing – review and editing. **Lukas Vaidelys:** Conceptualization; data curation; formal analysis; investigation; methodology; writing – original draft; writing – review and editing. **Julius Piluckis:** Data curation; investigation; resources. **Evaldas Padervinskis:** Data curation; formal analysis; resources; supervision. **Saulius Vaitkus:** Conceptualization; data curation; funding acquisition; resources; supervision. **Valdas Šarauskas:** Data curation; investigation; resources.

## FUNDING INFORMATION

None.

## CONFLICT OF INTEREST STATEMENT

There was no conflict of interest to declare.

## CONSENT

Written informed consent was obtained from the patient to publish this report following the journal's patient consent policy.

## Data Availability

The data that support the findings of this study are available from the corresponding author upon reasonable request.
